# Optimal hurricane overwash thickness for maximizing marsh resilience to sea level rise

**DOI:** 10.1002/ece3.2024

**Published:** 2016-03-25

**Authors:** David C. Walters, Matthew L. Kirwan

**Affiliations:** ^1^ Department of Physical SciencesVirginia Institute of Marine ScienceCollege of William and MaryPO Box 1346Gloucester PointVirginia23062

**Keywords:** Climate change, coastal geomorphology, disturbance event, ecosystem resiliency, press and pulse stresses, salt marsh, storm deposition

## Abstract

The interplay between storms and sea level rise, and between ecology and sediment transport governs the behavior of rapidly evolving coastal ecosystems such as marshes and barrier islands. Sediment deposition during hurricanes is thought to increase the resilience of salt marshes to sea level rise by increasing soil elevation and vegetation productivity. We use mesocosms to simulate burial of *Spartina alterniflora* during hurricane‐induced overwash events of various thickness (0–60 cm), and find that adventitious root growth within the overwash sediment layer increases total biomass by up to 120%. In contrast to most previous work illustrating a simple positive relationship between burial depth and vegetation productivity, our work reveals an optimum burial depth (5–10 cm) beyond which burial leads to plant mortality. The optimum burial depth increases with flooding frequency, indicating that storm deposition ameliorates flooding stress, and that its impact on productivity will become more important under accelerated sea level rise. Our results suggest that frequent, low magnitude storm events associated with naturally migrating islands may increase the resilience of marshes to sea level rise, and in turn, slow island migration rates. *Synthesis*: We find that burial deeper than the optimum results in reduced growth or mortality of marsh vegetation, which suggests that future increases in overwash thickness associated with more intense storms and artificial heightening of dunes could lead to less resilient marshes.

## Introduction

Ecosystem resiliency is determined by responses to a combination of long‐term press and short‐term pulse disturbances. The interplay can be complex, where pulse disturbances can either accelerate ecosystem change, or else enhance resiliency to long‐term stresses. Coastal ecosystems such as barrier islands and salt marshes face both long‐term stresses associated with sea level rise that threaten to drown low‐lying coastal landscapes, as well as short‐term disturbances from storms that result in pulses of inundation, erosion, and deposition. Climate change is projected to increase hurricane intensity as well as the rate of sea level rise (Vermeer and Rahmstorf 2009; IPCC 2014), making the interplay between these drivers complex and important to landscape evolution and ecosystem function.

Barrier islands and the marshes behind them play an important role in protecting valuable coastal property from storms. Including storm protection, recent work values ecosystem services provided by marshes at $10,000/ha (Barbier and Hacker 2011). Human populations on barrier islands are more dense than in their mainland states (Zhang and Leatherman 2011) and have grown at an accelerated rate that is faster than the rest of the nation (Culliton 1998). As low‐lying coastal landforms, climate change threatens barrier‐marsh systems, including the effects of sea level rise (Sallenger et al. 2012; IPCC 2014) and storms (Bender et al. 2010). The response of barrier island systems to climate change is also closely linked to human impacts (McNamara et al. 2011; Kirwan and Megonigal 2013), through processes such as shoreline hardening and artificial dune‐building which alter natural sedimentation processes. This creates a two‐way coupling whereby human behavior in response to coastal flooding and erosion can reduce ecosystem resiliency, thus diminishing the very ecosystem services that protect the human systems from flooding and erosion.

Traditional approaches study the response of barrier islands and marshes to climate change separately. However, recent numerical modeling proposes a fundamental coupling between the behavior of marshes and barriers (Lorenzo‐Trueba and Ashton 2014; Walters et al. 2014). For example, backbarrier fringe marshes provide a platform for barrier islands to migrate over that reduces the rate of island migration, while sand from the island helps to maintain narrow fringe marshes under conditions of suspended sediment supply and sea level rise that they would otherwise not be able to survive (Walters et al. 2014). Overwash, the transport of sand from the beach and dune to the backbarrier environment during storms, is the key process that links barrier islands and marshes. While episodic overwash provides an important source of sediment to build the backbarrier vertically (Dolan and Godfrey 1973), burial can also lead to plant mortality and decades‐long recovery (Osgood et al. 1995; Wang and Horwitz 2007). Although storms typically deposit sediment on marshes (Cahoon 2006; Tweel and Turner 2012), they can also cause substantial erosion (Cahoon 2006; Nikitina et al. 2014). Alternatively, the subsidy of sediment provided by overwash could add nutrients and help stave off the negative impacts of sea level rise on backbarrier marsh vegetation, thus leading to enhanced productivity (Mendelssohn and Kuhn 2003). Here, we study the impact of experimental hurricane overwash on marsh plant growth along an inundation gradient representative of sea level rise in an effort to understand how interacting facets of climate change will influence the coupled marsh‐barrier system. We find that shallow burial leads to enhanced plant productivity, but that deep burial leads to reduced growth and mortality, so that subtle changes in the frequency and magnitude of hurricane overwash will impact the resilience of marshes and barrier islands to long‐term sea level rise.

## Materials and Methods

### Site description

**Figure 1:**
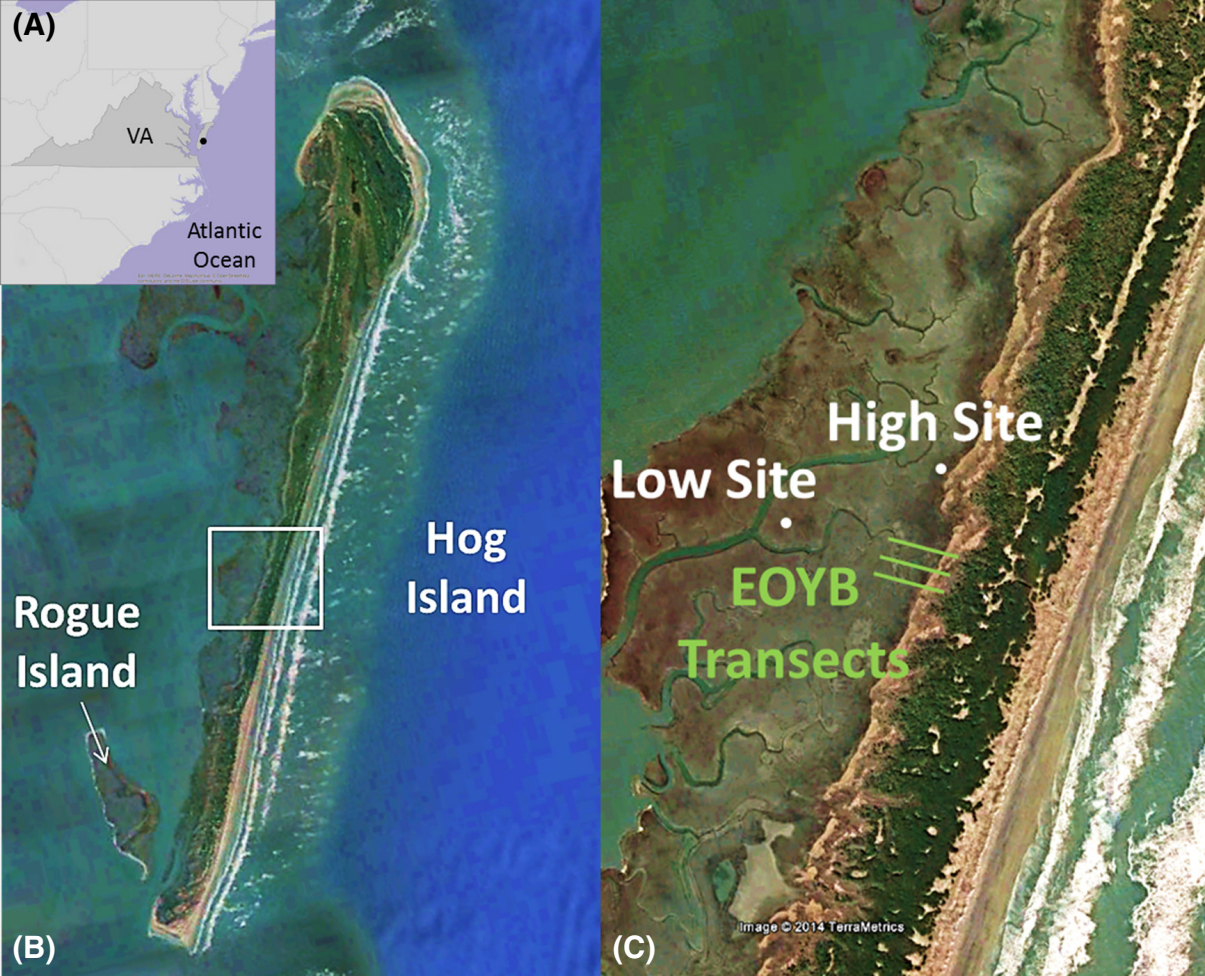
(A) Location of Hog Island study site on the Atlantic coast of Virginia (USA). (B) Google Earth photography illustrates extensive marsh behind a sandy barrier island with vegetated dunes and (C) the location of experimental plots relative to long‐term end of year biomasssampling transects.

We measured the response of marsh productivity to burial by experimental overwash at Hog Island, Virginia (USA) (Fig. 1). Hog Island is part of the VCR (Virginia Coast Reserve), an approximately 150 km barrier island coastline that evolves largely without human alteration. Local relative sea level rise rates exceed 3.0 mm year^−1^, where land subsidence and proximity to the Gulf Stream result in a mid‐Atlantic hot spot of accelerated sea level rise (Sallenger et al. 2012). Overwash of nutrient poor barrier island sand frequently buries adjacent tidal marshes and, in some cases, leads to vegetation mortality and re‐establishment that resembles primary succession (Osgood et al. 1995). Our experiments are located in or near a backbarrier fringe marsh that developed on an overwash fan associated with a storm in 1962 (Walsh 1998), and immediately adjacent to long‐term marsh biomass sampling sites associated with the VCR Long Term Ecological Research site (Fig. 1C).

To explore the combined influences of overwash and sea level rise, we conducted our experiment at a pair of low and high elevation marsh sites that are subject to different periods of tidal inundation and different rates of ambient plant productivity. Solinst Levelogger © pressure transducers at each location indicated that the low marsh site (15–20 cm NAVD) was flooded ~26% of the experiment duration, whereas the high marsh (50–55 cm NAVD) site was flooded ~9% of the time. Vegetation at both sites is predominantly *Spartina alterniflora*, with maximum plant heights of approximately 60 cm in the low marsh and 30 cm in the high marsh.

### Experimental design

**Figure 2:**
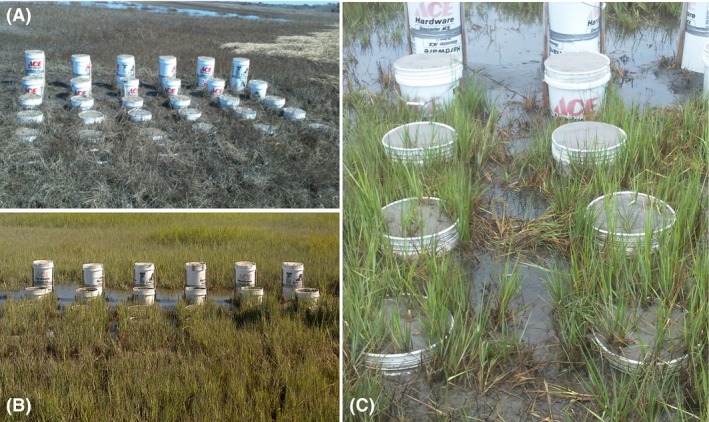
Experimental design at the high elevation marsh site showing replicate mesocosms filled with sand to depths of 0, 5, 10, 15, 30, and 60 cm. Photographs at (A) beginning and (B) end of the growing season illustrate rapid plant recovery. (C) Photograph illustrates decreasing above ground biomass with increasing burial depth, from 5 cm (front) to 30 cm (back).

The experiment simulated a range of burial depths (0–60 cm) representing literature derived overwash thicknesses that vary from 5 to 10 cm, which coincide with smaller storms with recurrence intervals of a couple years (Kochel and Dolan 1986), to more than 50 cm for larger storms with multidecadal recurrence intervals such as hurricanes (Foxgrover 2009; Eelkema et al. 2012). We constructed two arrays (one low marsh, one high marsh) that manipulate burial depth at a single point in order to isolate the effect of burial from other variables (e.g., salinity, underlying topography) that would be difficult to control for in a survey of natural overwash fans. Therefore, our approach provides a relatively quick assessment of the response of plant growth to overwash deposition where long‐term records are not available (Walters et al. 2014). Each array contained 36 mesocosms constructed from 5‐gallon plastic buckets with a 30 cm diameter, arranged into six rows of identical heights of 0, 5, 10, 15, 30, and 60 cm (Fig. 2). We cut the bottom out of each bucket, drilled holes in the side to enhance lateral drainage, and anchored them with wooden stakes so that approximately 1 cm of the bucket was buried below the marsh surface, thus varying the height of the bucket above the surface (Fig. 2). Experiments began prior to the growing season in March, 2014 to simulate storms such as winter Nor'easters and hurricanes that occur in the late summer and early fall of the previous year. Mesocosms of each height were filled with beach sand from a nearby source (Rogue Island, Fig. 1B) with a grain size of >99% sand, burying the pre‐existing vegetation below a layer of sand. The 0 cm row represented a control and consisted of the rim of the bucket staked down with no sand in it. We measured stem height and density approximately monthly, until the end of the growing season, August, 2014.

In this preliminary experiment designed to isolate the effect of burial on the growth response of an important marsh macrophyte, we have made several important assumptions. The most significant is that our experimental design approximates how plants will respond to burial by storm overwash events. We recognize, for example, that drainage in a mesocosm surrounded by plastic is likely different than in a natural marsh. We drilled holes in the side of the buckets and lined them with silt fencing material to allow drainage. The experiment also differs from natural overwash fans in that the spreading of plants from outside the mesocosm to inside via underground rhizomes can facilitate recovery faster than would occur in a natural fan with a larger areal extent. We buried the buckets 1 cm into the soil in an attempt to limit the spreading of shallow rhizomes into the mesocosm. Finally, shading and its effect on soil temperature could also influence plant growth in the experiments. We oriented the arrays so that each row faced south to minimize shading effects and to ensure that each row received the same exposure to sunlight.

### Laboratory and analytical methods

At the end of the experiment, we used destructive harvest techniques to measure plant productivity above, within, and below the sand layer in each mesocosm. Plant stems were clipped at the sand surface to isolate standing above‐sand layer material. Material within the sand layer was uncovered by removing the buckets and sand, and clipping the remaining vegetation at the original marsh surface. Potential differences in productivity below the sand layer were evaluated by taking 10 cm cores into the original marsh soil. All plant materials were transported to the laboratory and stored at 4^o^ C until processed. We sorted above‐sand layer material into live and dead fractions and sorted the within‐sand layer material into four components: dead, new growth shoots, old growth shoots, and roots (including all root material and rhizomes). We distinguished between shoots that existed prior to burial (old growth) and shoots that occurred after burial (new growth) based on diameter. We defined old growth as the shoot with the largest diameter within a group of shoots that were connected at the same internode, and each of the offshoots from that node are considered new growth. Both above and within‐sand layer material was rinsed to remove sediment and dried to a constant weight to record biomass. For aboveground material, this EOYB (end of year biomass) can be used as a proxy for productivity, following the standard method used annually to determine EOYB for the LTER (Long Term Ecological Research) long‐term record at the site (Christian and Blum). The sediment from the cores taken from below each bucket was additionally combusted at 600°C to determine percent organic matter (which includes organic matter from flora, fauna, and organic sediments) as a proxy for belowground production. To test the hypothesis that there is an optimal burial depth for enhancing *S. alterniflora* productivity, we fit a polynomial regression to EOYB versus depth of burial, excluding the points that result in an EOYB of zero, calculate the *R* ^2^, and compute a *P*‐value by transforming the correlation to create a *t* statistic with zero degrees of freedom. Additionally, we run a two‐sample *t*‐test to determine whether the populations of each mesocosm are significantly different from one another at the 95% confidence interval.

To draw a comparison between the effect of burial on productivity, which we measure directly, and the effect of sea level rise, we conducted an analysis of pre‐existing records of biomass and sea level. The long‐term biomass record comes from the VCR‐LTER site and includes EOYB of aboveground plant material every year since 1999 (Christian and Blum). We selected the EOYB of *S. alterniflora* taken from the low marsh portion of transects located on Hog Island adjacent to our study site (Fig. 1C). The sea level record comes from the NOAA National Data Buoy Center's nearest buoy to our site, located in Kiptopeke, VA, approximately 30 km south of Hog Island (NOAA 2015). We averaged the data across the months covering the *S. alterniflora* growing season (June–August) for each year and detrended by the long‐term linear rate of sea level rise (3.60 mm year^−1^) to calculate the SLA (sea level anomaly). To test the hypothesis that plant productivity is diminished in years of anomalously high sea level, we calculate a correlation coefficient between long‐term EOYB and SLA, and compute a *P*‐value by transforming the correlation to create a *t* statistic with zero degrees of freedom.

## Results

**Figure 3:**
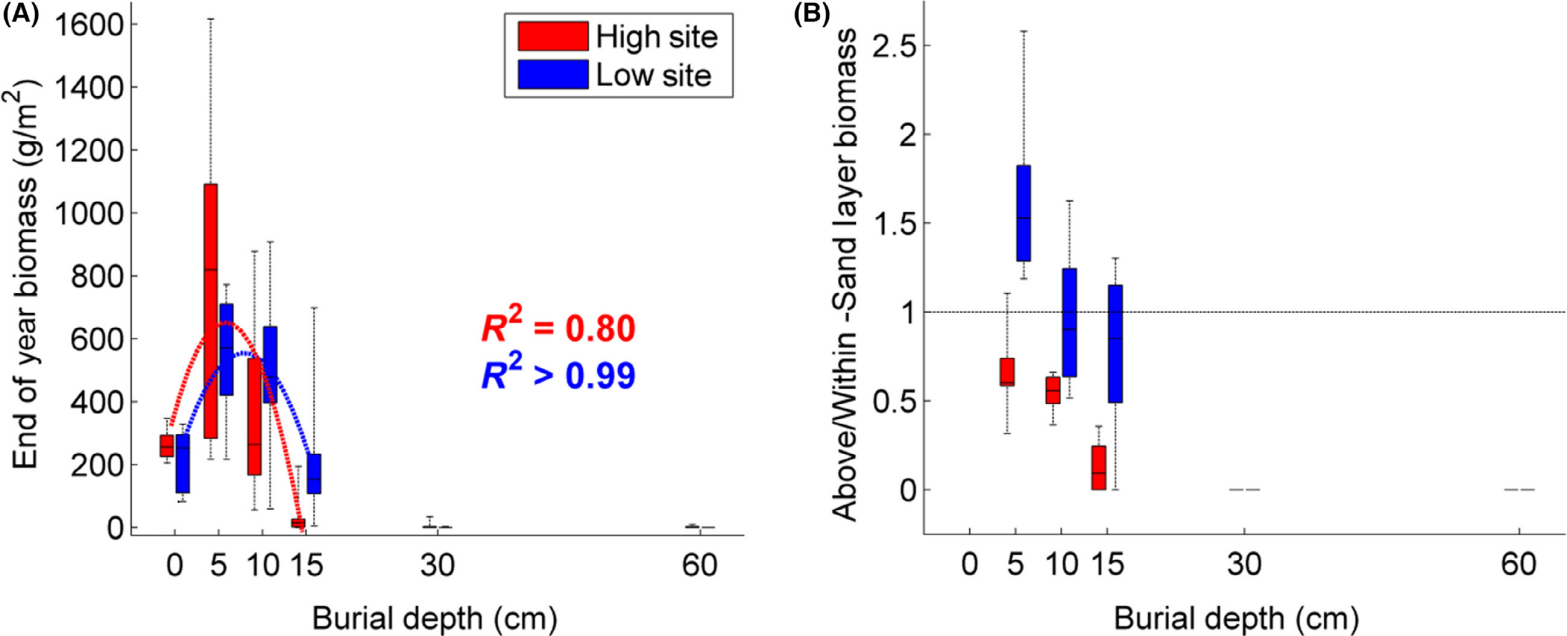
(A) Box and whisker plot of end of year biomass versus burial depth for each experimental treatment. Biomass represents the total living biomass above and within the sand layer across each replicate burial depth. Dashed lines represent best quadratic fits for high site (*y* = −0.61*x* ^2^ + 7.57*x* + 22.59, *R* ^2^ = 0.80, *P* = 0.11) and low site (*y* = −0.42*x* ^2^ + 6.21*x* + 16.10, *R* ^2^ > 0.99, *P* < 0.01) (B) Box and whisker plot of the ratio of above‐sand layer biomass to within‐sand layer biomass.

Both above‐sand layer and within‐sand layer components of *S. alterniflora* growth varied strongly as a function of burial depth, with total EOYB ranging from 15 to 350 g per mesocosm (~35–850 g m^−2^). Total EOYB followed a parabolic relationship with burial depth, with maximum biomass for the 5 cm sand addition treatments at both the low and high marsh sites (Fig. 3A). A best polynomial fit for each site gives a quadratic curve with maximum EOYB values at 6.2 cm for the high site and 7.4 cm for the low site (Fig. 3A). From two‐sample *t*‐tests, the 5 cm treatments at both sites had significantly greater EOYB compared to the control treatment for each site, but neither differed significantly from the 10 cm treatment. Therefore, we conclude that both the high and low site had a maximum EOYB at 5–10 cm. There was no plant growth in the 30 and 60 cm sand addition treatments, where deep burial led to plant mortality. Total biomass at the high elevation site was greater than the low site in the control and 5 cm mesocosms but less than the low marsh site in the 10 and 15 cm mesocosm. These results are supported by the quadratic fits for each group, which show that the biomass curve for the low elevation site is shifted to the thicker burial side of the plot, suggesting that the optimum burial depth is greater for the low site and potentially may increase with flooding frequency (Fig. 3A).

**Figure 4:**
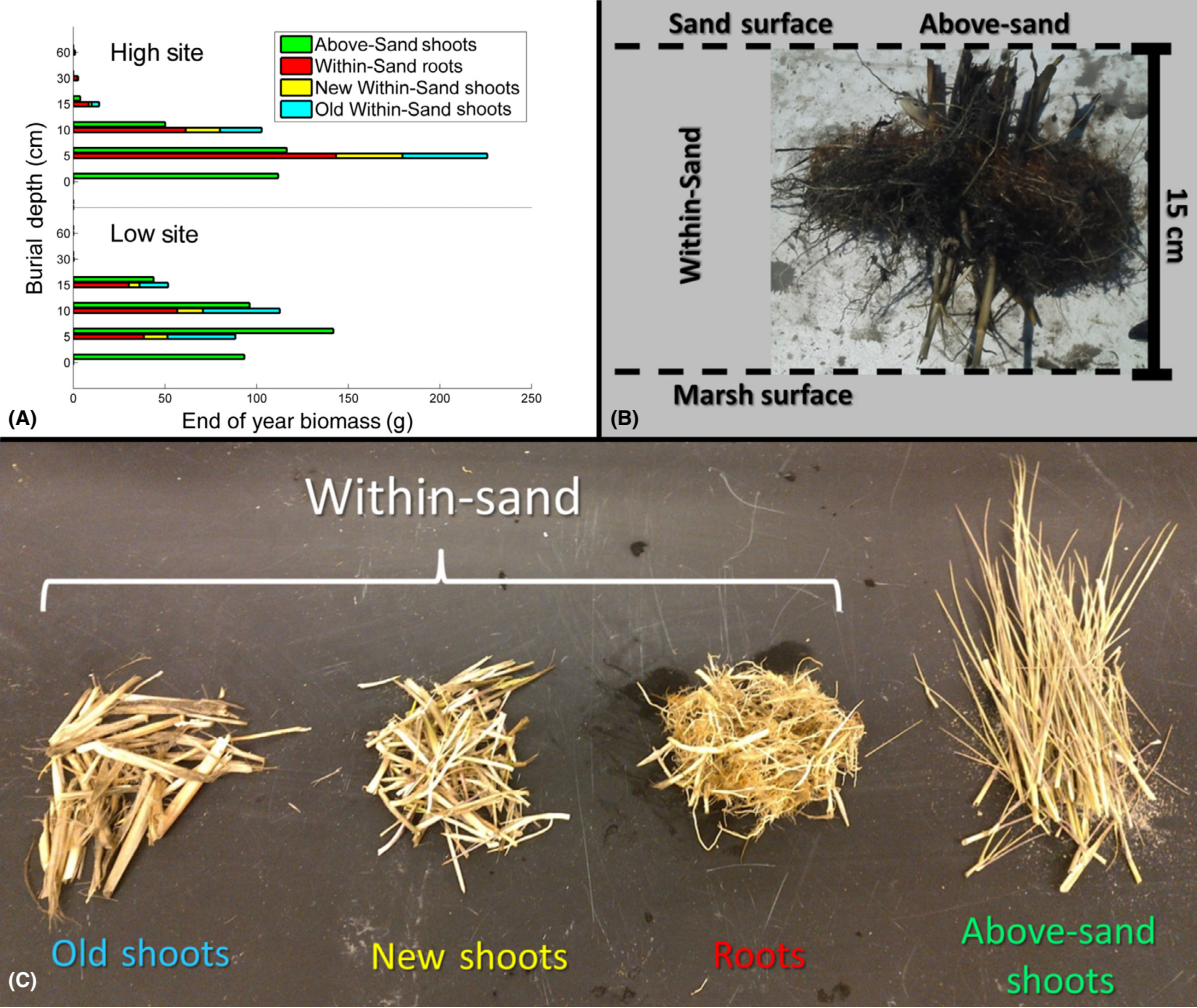
(A) Bar plot of end of year biomass for each burial depth treatment, broken down into its four components: above‐sand layer shoots, within‐sand layer roots, new within‐sand layer shoots, and old within‐sand layer shoots. The within‐sand layer components of biomass are stacked to show the total within‐sand layer biomass. (B) Picture of within‐sand layer plant material from a 15‐cm burial treatment at the low site, after being excavated at the end of the growing season. (C) Picture of plant material from a 5‐cm burial treatment at the high site sorted into above‐sand layer and within‐sand layer components.

Biomass within the sand layer comprised the dominant contributor to total biomass in most treatments, and the ratio between above‐ and within‐sand layer biomass decreased with burial depth (Fig. 3B). Maximum above/within‐sand layer biomass ratios were <1.6 for the low marsh site and <0.5 at the high marsh site (Fig. 3B). The primary component of the biomass within the sand layer was root growth, especially at the high site where adventitious root growth into the sand layer was greater than above‐sand layer growth for each burial depth (Fig. 4A). These roots developed directly from buried stems and accounted for 60–63% and 44–59% of the total biomass within the sand layer at the high and low elevation sites, respectively (Fig. 4A). Percent organic matter for the shallow marsh cores below each mesocosm ranged 5–8% and did not vary significantly with burial depth, suggesting little difference in the belowground production of plants below the original marsh surface.

## Discussion

Our results are consistent with a well‐established positive relationship between productivity and shallow marsh burial. From previous studies of hurricane deposits, a deposition of 5–10 cm was found to lead to an increase in aboveground *S. alterniflora* productivity of 20–60% (Baustian and Mendelssohn 2015) and a deposition of 3–8 cm was found to cause an ~50% increase in aboveground marsh plant biomass (McKee and Cherry 2009), which matches well with the ~50% increase in aboveground biomass we observed at the low site for a burial depth of 5 cm (Fig. 4A). Additionally, studies of dredge spoil deposits (Wilber 1993; Ford et al. 1999; Mendelssohn and Kuhn 2003) and experimental sediment additions (Fragoso and Spencer 2008) have found a similar positive relationship between burial and marsh plant productivity. Recent work suggests that the mechanism for increased primary productivity associated with burial could be related to an increase in nutrient levels from ammonium and phosphate that is sorbed to the clay fraction of the deposited sediments (Baustian and Mendelssohn 2015). This explanation may not be applicable for our study, where the sediment added was >99% sand from a nutrient poor barrier island beach environment (Osgood and Zieman 1993). Alternatively, the sediment subsidy hypothesis suggests that sediment deposition can increase soil aeration and slow phytotoxin accumulation, thus allowing plant roots to avoid alcoholic fermentation which inhibits growth (Mendelssohn et al. 1981; Mendelssohn and Kuhn 2003).

Our results are also consistent with previous work that shows that sediment addition (8–23 cm) leads to the formation of new roots and shoots emerging from plant rhizomes (Wilber 1993) or basal meristems (Fragoso and Spencer 2008) buried just below the new soil surface. We observed a similar development of a new root system within the deposited sand layer (Fig. 4B) that resulted in typical above‐ to within‐sand layer biomass ratios of less than 1 (Fig. 3B). This demonstrates the relative importance of underground growth as a mechanism for plant recovery in response to burial and suggests that studies that only use measurements of aboveground biomass as a proxy for plant response to burial (Mendelssohn and Kuhn 2003; Baustian and Mendelssohn 2015) likely underestimate the level of recovery. We did not observe a root mat within the sand layer of the 30 and 60 cm burial treatments. We assume based on these results that these plants were unable to produce viable new shoots from their rhizomes. In order for recovery to take place without offshoots from pre‐existing rhizomes, new plants would have to grow from seeds, which is a slower process of recovery (Wilber 1993).

While most recent work highlights the positive effect of hurricane‐deposited sediment on aboveground productivity and resilience to sea level rise (McKee and Cherry 2009; Baustian and Mendelssohn 2015), our results indicate that there is an optimum burial thickness for enhanced plant biomass. The large (40–120%) increase in productivity we observe is maximized for burial depths of 5–10 cm, while burial greater than or equal to 30 cm resulted in mortality and no recovery (Fig. 3A). Previous work concluding that there is a positive linear relationship between overwash deposition and plant productivity is limited to deposition events less than 12 cm thick (Baustian and Mendelssohn 2015) and therefore would be unable to discern the nonlinear relationship associated with greater burial depths. Interestingly, our observed optimum burial depth varies for marshes of different elevations, where low elevation marshes likely have a higher optimal burial depth (7.4 cm) than high elevation marshes (6.2 cm) (Fig. 3A). We attribute this variability to differences in flooding and the impact of redox potential on vegetation recovery following burial (Croft et al. 2006). This has relevance for wetlands restoration efforts that use sediment additions to combat sea level rise, as it provides a method for determining the site‐specific optimal burial depth which could lead to more efficient restoration efforts.

Other studies have observed a similar unimodal trend in marsh plant response to sea level rise. For example, *Pucinellia maritima*, a common salt marsh grass in the Bay of Mont Saint Michael in France, shows stimulated above‐ and below‐ground biomass in response to 4 mm of burial per month, reduced growth with 8 mm of burial per month, and mortality at a rate of 12 mm burial per month (Langlois et al. 2001). Results from sediment slurry additions, where a mixture of water and dredged sediment is added to degrading marshes for restoration purposes, also show a threshold amount of burial, past which point there is a decrease in ecosystem resiliency (Stagg and Mendelssohn 2011) and a reduction in above‐ and below‐ground net primary productivity, although not complete mortality (Stagg and Mendelssohn 2010). Our results are in line with these findings of reduced ecological function in areas of excessive sediment addition. However, in the case of the sediment slurry additions, the effect of elevation and burial is conflated, because the studies are conducted several years after the sediment additions. Thus, the results do not capture the immediate effect of burial on marsh plants and instead are more influenced by the resulting elevation gradient and its effect on inundation, which also shows a unimodal response.

**Figure 5:**
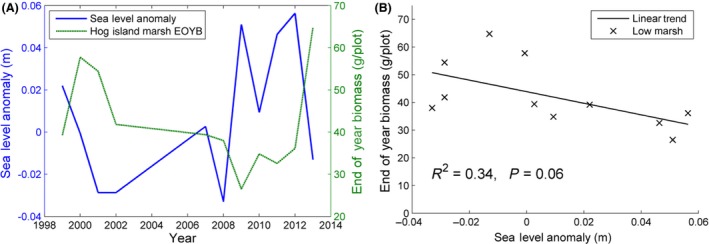
(A) SLA (Sea Level Anomaly) and EOYB (End of Year Biomass) as they co‐vary through time. SLA shows the deviation from the long‐term linear trend of sea level rise. EOYB is a measurement of *Spartina alterniflora* aboveground biomass from the end of the growing season, taken annually at the VCR‐LTER's long‐term biomass monitoring site on Hog Island. There is an inverse trend between *S. alterniflora* productivity and sea level. (B) EOYB from the VCR‐LTER versus SLA, showing significant (*P* < 0.1) downward trend in *S. alterniflora* productivity with increasing sea level.

Hurricanes are well known to provide an important source of sediment to marshes that may otherwise be unable to keep pace with sea level rise (Cahoon et al. 1995; Nyman et al. 1995; Turner et al. 2006; McKee and Cherry 2009). However, the effect of that sediment deposition on the ecological function of marsh vegetation is more poorly understood. Excessive inundation and soil saturation limits marsh productivity in low elevation marshes and in regions of rapid sea level rise (Mendelssohn and McKee 1988; Pezeshki et al. 1991; Kirwan and Guntenspergen 2012). Therefore, storms have the potential to increase marsh resiliency to sea level rise by increasing their elevation relative to sea level and increasing the aeration of the soil (Mendelssohn and Kuhn 2003). Long‐term biomass measurements at our study site indicate that production is lowest in years of high sea level, where a positive sea level anomaly of ~10 cm corresponds to an ~40% reduction in biomass (Fig. 5B). Meanwhile, our experimental results suggest that a 10 cm increase in elevation can lead to an ~80% increase in biomass (Fig. 3A). The magnitude of this effect is dependent on the initial elevation of the salt marsh (~40% increase for the high marsh and ~125% for the low marsh), suggesting that sediment deposition from storms is more effective at ameliorating flooding stress in low elevation marshes, and by inference, marshes in regions of rapid sea level rise.

As land managers, humans often serve as low‐pass filters on landscapes, eliminating more frequent low magnitude events that tend to benefit ecosystems (e.g., fires, floods, storms), while still allowing infrequent, larger magnitude disturbance events to occur (Werner and Mcnamara 2007). On barrier islands, for example, humans build artificial dunes that prevent overwash and gradual island migration, until a large storm overtops the artificially high dunes and the barrier morphology adjusts rapidly (Magliocca et al. 2011). Our results indicate that small amounts of overwash deposited on backbarrier marsh platforms (5–15 cm thickness) can enhance marsh productivity and resilience to sea level rise, but that larger deposits (≥30 cm) will lead to a decrease in marsh productivity and therefore ecosystem function. Thus, our results suggest that connectivity between barrier and marsh systems is a key aspect of system resilience to storms and sea level rise. Frequent, small overwash events that occur in natural barrier systems lead to enhanced rates of marsh productivity and vertical accretion (Nyman et al. 1995), which in turn can help to slow the rate of island migration (Walters et al. 2014). In contrast, anthropogenic barriers to overwash lead to infrequent, high magnitude events that we show would be detrimental to marsh productivity, and may therefore reverse the natural tendency for storms to increase marsh and barrier island resilience.

## Data Accessibility

Data will be uploaded to VCR‐LTER data catalog and assigned a DOI at the time of manuscript acceptance.

## Conflict of Interest

None declared.
